# Up-regulation of endothelin type B receptors in the human internal mammary artery in culture is dependent on protein kinase C and mitogen-activated kinase signaling pathways

**DOI:** 10.1186/1471-2261-8-21

**Published:** 2008-09-08

**Authors:** David Nilsson, Lotta Gustafsson, Angelica Wackenfors, Bodil Gesslein, Lars Edvinsson, Per Paulsson, Richard Ingemansson, Malin Malmsjö

**Affiliations:** 1 Department of Medicine, Lund University Hospital, Sweden; 2 Department of Ophthalmology, Lund University Hospital, Sweden; 3 Department of Cardiothoracic Surgery, Lund University Hospital, Sweden

## Abstract

**Background:**

Up-regulation of vascular endothelin type B (ET_B_) receptors is implicated in the pathogenesis of cardiovascular disease. Culture of intact arteries has been shown to induce similar receptor alterations and has therefore been suggested as a suitable method for, *ex vivo*, in detail delineation of the regulation of endothelin receptors. We hypothesize that mitogen-activated kinases (MAPK) and protein kinase C (PKC) are involved in the regulation of endothelin ET_B _receptors in human internal mammary arteries.

**Methods:**

Human internal mammary arteries were obtained during coronary artery bypass graft surgery and were studied before and after 24 hours of organ culture, using *in vitro *pharmacology, real time PCR and Western blot techniques. Sarafotoxin 6c and endothelin-1 were used to examine the endothelin ET_A _and ET_B _receptor effects, respectively. The involvement of PKC and MAPK in the endothelin receptor regulation was examined by culture in the presence of antagonists.

**Results:**

The endohtelin-1-induced contraction (after endothelin ET_B _receptor desensitization) and the endothelin ET_A _receptor mRNA expression levels were not altered by culture. The sarafotoxin 6c contraction, endothelin ET_B _receptor protein and mRNA expression levels were increased after organ culture. This increase was antagonized by; (1) PKC inhibitors (10 μM bisindolylmaleimide I and 10 μM Ro-32-0432), and (2) inhibitors of the p38, extracellular signal related kinases 1 and 2 (ERK1/2) and C-jun terminal kinase (JNK) MAPK pathways (10 μM SB203580, 10 μM PD98059 and 10 μM SP600125, respectively).

**Conclusion:**

In conclusion, PKC and MAPK seem to be involved in the up-regulation of endothelin ET_B _receptor expression in human internal mammary arteries. Inhibiting these intracellular signal transduction pathways may provide a future therapeutic target for hindering the development of vascular endothelin ET_B _receptor changes in cardiovascular disease.

## Background

Endothelin-1 is a potent vasoconstrictor produced by endothelial cells. It is a vasoactive agent that mediates multiple vascular actions and plays an important role in hypertension and cardiovascular diseases by promoting changes in vascular reactivity and endothelial function, cardiovascular fibrosis, tissue remodeling, inflammation, and oxidative stress. Endothelin exerts its effect through two different G protein coupled receptors, the endothelin type A (ET_A_) receptor and the endothelin type B (ET_B_) receptor [1-3]. The endothelin ET_A _receptors are expressed in vascular smooth muscle cells and mediate vasoconstriction. In healthy conditions, endothelin ET_B _receptors are mainly located on endothelial cells and mediate vasodilatation via the release of nitric oxide, prostaglandins and endothelium-derived hyperpolarizing factor [4-6]. However, endothelin ET_B _receptors on vascular smooth muscle cells have been observed to be upregulated during pathological conditions such as atherosclerosis [7], congestive heart failure [8], ischemic heart disease [9] and hypertension [10]. Endothelin receptors on vascular smooth muscle cells are both mitogenic, leading to atherosclerosis, and mediate strong vasoconstriction which may lead to elevated vascular tone frequently observed in cardiovascular disease.

Endothelin receptor regulation can be studied in detail, *ex vivo*, using organ culture of intact arteries. Endothelin ET_B _receptors on smooth muscle cells are up-regulated when whole blood vessels are incubated for 12 to 48 hours [11]. Furthermore, endothelin ET_B _receptors are up-regulated in human coronary arteries after organ culture, in a similar way as in ischemic heart disease in man [12]. Endothelin receptor-changes also occur during organ culture in rat cerebral and peripheral arteries, mimicking that observed in peripheral artery disease, stroke and subarachnoidal haemorrhage [13-15]. Detailed delineation of the regulation of vascular endothelin receptors can be performed by culture in the presence of different humoral factors or intracellular signal transduction pathway inhibitors.

We aim to identify the intracellular signal transduction pathways that regulate the expression of endothelin receptors in the vasculature. These may provide future therapeutic targets for hindering the development of vascular endothelin receptor changes in cardiovascular disease. In a previous study, culture of porcine coronary arteries shows that protein kinase C (PKC) and mitogen activated protein kinases (MAPKs) are signaling pathways that regulate endothelin receptor expression [16]. Other studies, using rat cerebral arteries, show similar results [17,18]. Hitherto, the regulation of endothelin receptors have mainly been studied in animals and data from humans barely exists. When identifying new targets for pharmaceutical intervention, it is of importance that the research is performed not only in animals, but also in patients. In the present study, internal mammary arteries from patients undergoing coronary artery bypass graft surgery were studied to examine the role of PKC and MAPK in the endothelin ET_A _and ET_B _receptor regulation in humans.

PKC is a family of serine/threonine kinases participating in signal transduction events in response to specific hormonal, neuronal and growth factor stimuli. MAPKs represent another group of serine/threonine kinases that are thought to act downstream from PKC in the smooth muscle cell regulatory cascade [19]. There are three major groups of distinctly regulated MAPKs leading to altered gene expression in humans. The extracellular signal related kinases 1 and 2 (ERK1/2), the C-jun terminal kinase (JNK) and the p38 MAPK are known to play important roles in the intracellular signalling in response to extracellular stimuli [20]. Upon activation, MAPKs cause phosphorylation and activation of transcription factors present in the cytoplasm or nucleus, thereby leading to expression of target genes resulting in biological responses [21].

In the present study, we use the organ culture model to examine the involvement of PKC and MAPK pathways in the regulation of endothelin receptors in the human internal mammary artery. Arterial segments are cultured for 24 hours in the absence or presence of PKC inhibitors (Ro-32-0432 and bisindolylmaleimide I) and inhibitors of the three major MAPK pathways in mammals (p38 MAPK, ERK1/2 and JNK). The contractile effects and the levels of endothelin ET_A _and ET_B _receptor protein and mRNA expression are evaluated using *in vitro *pharmacology, real time PCR and Western blot techniques.

## Methods

### Tissue collection

During coronary artery bypass graft surgery, one end of the left mammary artery is harvested and sutured to the coronary artery, distal to the stenosis. A segment of the artery is sometimes removed when the artery is adjusted to an appropriate length for the grafting procedure. The artery segment that is removed can be used for research without affecting the patient. For the present study, the left internal mammary artery, from 27 patients undergoing coronary artery bypass graft surgery, was used for experimental analysis. The patients' median age was 70 years and ranged from 43 to 85 years. Twenty-one men and 6 women were included in the study. After dissection during surgery, the vessels were immediately immersed into cold sterile Dulbeccos' modified Eagles' medium (DMEM), transported to the laboratory on dry ice and used for the experiments. In the laboratory, the arteries were dissected free from adhering tissue, and then cut into cylindrical segments (3–4 mm long).

### Ethics

The study was approved by the Ethics Committee of Lund University in Sweden and is in accordance with the Declaration of Helsinki.

### Organ culture procedure

The artery segments were divided into two groups; one that was not cultured (control) and one that was cultured for 24 hours. The artery segments for culture were placed in a 48 well plate, one segment in each well, containing 1 ml DMEM and incubated for 24 hours at 37°C in humidified 5% CO_2 _in air. DMEM (Gibco BRL, Praisley, UK) was serum free and contained D-glucose (1 g/l), sodium pyruvate (100 mg/l) and was supplemented with penicillin (100 U/ml), streptomycin (100 μg/ml) and amphotericin B (0.25 μg/ml). The method of blood vessel culture has been described previously [11]. The segments were cultured in the absence or presence of:

#### (1) PKC inhibitors

• Ro-32-0432 (10 μM), 2-8-[(Dimethylamino)methyl]-6,7,8,9-tetrahydropyrido [1,2-a]indol-3-yl}-3-(1-methyl-1H-indol-3-yl)maleimide.

• Bisindolylamaleimide I (10 μM), 2-[1-(3-Dimethylaminopropyl)-1H-indol-3-yl]-3-(1H-indol-3-yl)-maleimide.

#### (2) MAPK inhibitors

• The p38 MAPK inhibitor, SB 203580 (10 μM), 4-(4-Fluorophenyl)-2-(4-methylsulfinylphenyl)-5-(4-pyridyl)-1H-imidazole.

• The ERK1/2 inhibitor, PD 98059 (10 μM), 2-(2-Amino-3-methoxyphenyl)-4H-1-benzopyran-4-one.

• The JNK inhibitor, SP600125 (10 μM), 1,9-Pyrazoloanthrone Anthrapyrazolone.

### *In vitro *pharmacology

For the *in vitro *pharmacology experiments, the arterial segments were mounted on two L-shaped metal prongs, one of which was connected to a force displacement transducer for continuous recording of the isometric tension [22]. The mounted segments were immersed in temperature controlled (37°C) tissue baths containing a bicarbonate based buffer solution of the following composition; NaCl (119 mM), NaHCO_3 _(15 mM), KCl (4.6 mM), MgCl (1.2 mM), NaH_2_PO_4 _(1.2 mM), CaCl_2_, (1.5 mM) and glucose (5.5 mM), which was continuously gassed with 5 % CO_2 _in O_2 _resulting in a pH of 7.4. Eight to sixteen segments were studied at the same time in separate tissue baths. The segments stabilized at a resting tension of 4 mN for one hour before the experiments were started. Previous results show that a resting tension of 3 to 5 mN provides optimal conditions for studying vascular contraction in the human left internal mammary artery [23]. The contractile capacity of each arterial vessel segment was examined by exposure to a potassium rich (63.5 mM) buffer solution.

The endothelin ET_B _receptor agonist, sarafotoxin 6c, was first added at increasing concentrations (10^-11^-10^-6 ^mM). The arteries were washed and endothelin-1 was therafter added at increasing concentrations (10^-11^-10^-6 ^mM). At this stage the endothelin ET_B _receptors were desensitized [24], allowing endothelin-1 to act selectively on endothelin ET_A _receptors.

The sarafotoxin 6c experiments were run in the absence (control) and presence of the selective endothelin ET_B _receptor antagonist BQ788 ((N-cis-2,6-dimethyl-piperidinocarbonyl-L-γ-methylleucyl-D-1-methoxycarbonyltryptophanyl-D-norleucine, 0.1 μM), added 15 min prior to sarafotoxin 6c.

Previous results from human internal mammary arteries show a variation in the expression of the vasoconstricting endothelin ET_B _receptors and only 58 % of the patients that undergo coronary artery bypass graft surgery have graft vessels that express these receptors [23]. Other studies have shown similar irregularity in the endothelin response [25]. In the present study, 44 % of the examined arteries (patients) responded to sarafotoxin 6c. For the *in vitro *pharmacology experiments, using BQ788, only the arteries that responded to sarafotoxin 6c was used. For the other experiments, both the arteries that responded and the arteries that did not respond to sarafotoxin 6c were used for the experiments, calculations and results.

All drugs for the *in vitro *pharmacological experiments were purchased from Sigma Chemical Co (St. Louis, MO). Endothelin-1 and sarafotoxin 6c were dissolved in 0.9 % NaCl with 10 % albumin and BQ788 were dissolved in 0.9 % saline. The PKC and MAPK inhibitors were dissolved in dimethylsulphoxide (0.01 M DMSO in 0.9 % saline, Calbiochem ^®^, Darmstadt, Germany).

### Real time PCR

The arteries for real time PCR experiments were frozen in liquid nitrogen and stored at -80°C until the experiments were performed. Endothelin ET_A _and ET_B _receptor mRNA expression levels were quantified by real time PCR. Total cellular RNA was extracted using TRIzol^®^LS according to the supplier's instructions (Life Technologies, Paisley, UK). Reverse transcription of total RNA to cDNA was carried out using the Gene Amp RNA PCR kit in a DNA Thermal cycler (Perkin-Elmer Applied Biosystems, Foster City, CA, USA). Real time PCR was performed with the Gene-Amp SYBR Green PCR kit (PE Applied Biosystems) in a Perker-Elmer real time PCR machine 7300.

The cDNA synthesized above served as template in a (25 μl) reaction. A non-template control was included in all experiments. The GeneAmp 7300 sequence detection system monitored the binding of a fluorescent dye to double-strand DNA by real time detection of the fluorescence during each cycle of PCR amplification. Specific primers were designed as follows:

ET_A _receptor GenBank: NM_001957 forward; 5'-ATTGCCCTCAGCGAACAC-3' reverse; 5'-CAACCAAGCAGAAAGACGGTC-3' ET_B _receptor GenBank: NM_000115 forward; 5'-GATACGACAACTTCCGCTCCA-3' reverse; 5'-GTCCACGATGAGGACAATGAG-3' β-actin GenBank: NM_001101 forward; 5'-AAGGCCAACCGCGAGAA-3' reverse; 5'-ACAGCC TGGATAGCAACGTACA-3' GAPDH GenBank: NM_002046 forward; 5'-CACCAGGGCTGCTTT TAACTCT-3' reverse; 5'-CTTGACGGTGCCATGGAATT-3'

The housekeeping genes, β-actin and glyceraldehyde 3-phosphate dehydrogenase (GAPDH) were used as references due to their continuous expression in cells. The real time PCR reaction was performed at a temperature of 50°C for 2 min, 95°C for 10 min, and the following 40 PCR cycles with 95°C for 15 s and 60° for 1 min. Oligonucleotides and reagents for the PCR assay were purchased from Perkin-Elmer, Applied Biosystems Foster City, CA, USA.

### Western Blot

#### Preparation of cell lysates

Whole cell extracts from the human internal mammary arteries were prepared by adding 300 μl of RIPA buffert (50 mM Tris, pH 8.0, 1.0% Igepal CA-630, 0.5% sodium deoxycholate, 0.1% SDS, 150 mM NaCl) supplemented with 0.37 g/ml Complete protease inhibitor cocktail (Roche Diagnostics, Mannheim, Germany). By using a Tissue Lyser (Retsch GmbH, Haan, Germany) the samples were homogenized for 3 minutes at maximum frequency. Thereafter, the samples were incubated for 2 hours under gentle rocking at 4°C, where after the samples were centrifuged at 12 000 g for 20 min and the supernatant was collected for protein concentration determination (Protein Assay Dye, Bio Rad, CA, USA).

#### Experimental procedure

Cell extracts were denatured in LDS sample buffer for 5 min in 95°C, run on SDS-PAGE (NUPAGE, 4–12% Bis-Tris, Invitrogen, Carlsbad, CA, USA) and blotted onto PVDF membrabes (0.2 μm, Invitrogen). Membranes were blocked with 2% non-fat dried milk for 1 hour and incubated with 1:100 goat polyclonal antibodies to human ET_B _receptor (C-20, sc-21196, Santa Cruz Biotechnology, Santa Cruz, Ca, USA) and 1:1000 HRP-coupled donkey anti-goat secondary antibody (DakoCytomation, Glostrup, Denmark). The membrane was developed by using the ECL Plus Western Blotting Reagent (GE Healthcare, Little Chalfont, UK) and Fuji Film LAS-1000 equipment (Fuji Film, Tokyo, Japan). Parallel membranes were incubated with 1:5000 mouse monoclonal antibodies to beta-actin (C4, sc-47778, Santa Cruz Biotechnology) and HRP-coupled rabbit anti-mouse secondary antibody (DakoCytomation). Primary and secondary antibody solutions were prepared in PBS solution containing 2% bovine serum albumin 0.1% Tween-20. After incubation with antibodies, the membranes were washed 3 times and 5 min in PBS containing 0.1% Tween-20.

#### Calculations and statistics

Calculations and statistics were performed using Graph Pad 4.0 software. Statistical analysis was performed using Student's t-test when comparing two groups and ANOVA with Dunnett's post-test for multiple comparisons when comparing three groups or more. P < 0.05 was considered significant. The results are expressed as mean ± standard error of the mean (S.E.M.).

##### *In vitro *pharmacology

The maximum contraction (E_max_) was calculated as percentage of the contractile capacity of 63.5 mM potassium. The negative logarithm of the concentration that elicited 50% contraction (pEC_50_) was determined by linear regression analysis using the values immediately above and below half-maximum response.

##### Real time PCR

The amount of endothelin ET_A _and ET_B _receptor mRNA expression was calculated as relative to the amount GAPDH or β-actin in the same sample by the formula X_0_/R_0 _= 2CtR-CtX, where X_0 _= amount of endothelin ET_B _mRNA, R_0 _= original amount of GAPDH or β-actin mRNA, CtR = Ct value for GAPDH or β-actin and CtX = Ct value for the endothelin ET_A _and ET_B _receptor mRNA.

## Results

### Effects of organ culture on endothelin ET_A _and ET_B _receptors

The endothelin ET_B _receptor mediated contraction was studied using the selective agonist sarafotoxin 6c. The endothelin-1-induced vasoconstriction was studied after desensitizing the endothelin ET_B _receptors with sarafotoxin 6c prior to adding endothelin-1, leaving only endothelin ET_A _receptors to respond.

**Figure 1:**
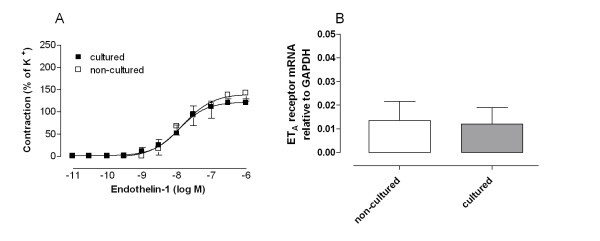
**(A) Endothelin-1 contractions (after ET_B _receptor desensitisation) and (B) ET_A _receptor mRNA levels in cultured and non-cultured human internal mammary arteries, examined using *in vitro *pharmacology (n = 27) and real time PCR (n = 8) experiments.** The results are shown as mean values ± S.E.M. Statistical analyses, comparing cultured with non-cultured, were performed using Student's t-test. There were no significant differences.

Endothelin-1 induced potent contractions in the human internal mammary arteries studied (Fig. 1). *In vitro *pharmacology and real-time PCR experiments demonstrated similar endothelin-1 contractions and endothelin ET_A _receptor mRNA levels before and after organ culture (P = n.s., n = 27 and 8 respectively, Fig. 1).

**Figure 2:**
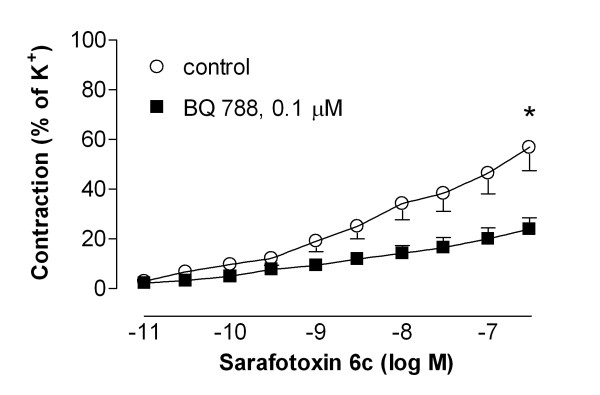
**Contractile responses elicited by cumulative application of sarafotoxin 6c in non-cultured segments of human internal mammary arteries in the absence (control) and presence of 0.1 μmol/l BQ788.** The results are shown as mean values ± S.E.M of six experiments. Statistical analyses of the maximum contraction, comparing control with BQ 788, was performed using Student's t-test, where P < 0.05 (*) was considered significant.

The endothelin ET_B _receptor agonist, sarafotoxin 6c, induced contraction in 44 % of the left internal mammary arteries (patients) studied (n = 27). The sarafotoxin 6c contraction was inhibited by the selective endothelin ET_B _receptor antagonist, BQ788 (P < 0.05, n = 6, Fig. 2). Both the arteries that responded and the arteries that did not respond to sarafotoxin 6c were used for further experiments, as described below.

**Figure 3:**
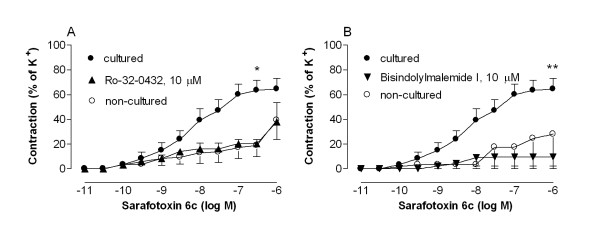
**Contractile responses elicited by cumulative application of the endothelin ET_B _receptor agonist sarafotoxin 6c in segments of human internal mammary arteries.** The arterial segments were either not cultured or cultured in the absence or presence of the protein kinase C inhibitors (A) Ro-32-0432 or (B) bisindolylmaleimide I. The results are shown as mean values ± S.E.M of six experiments. Statistical analysis was performed using ANOVA with Dunnett's post-test for multiple comparisons. P < 0.05 (*) and P < 0.01 (**) was considered significant. Comparisons were made between the results from arteries exposed to culture with and without Ro-32-0432 or bisindolylmaleimide I.

**Figure 4:**
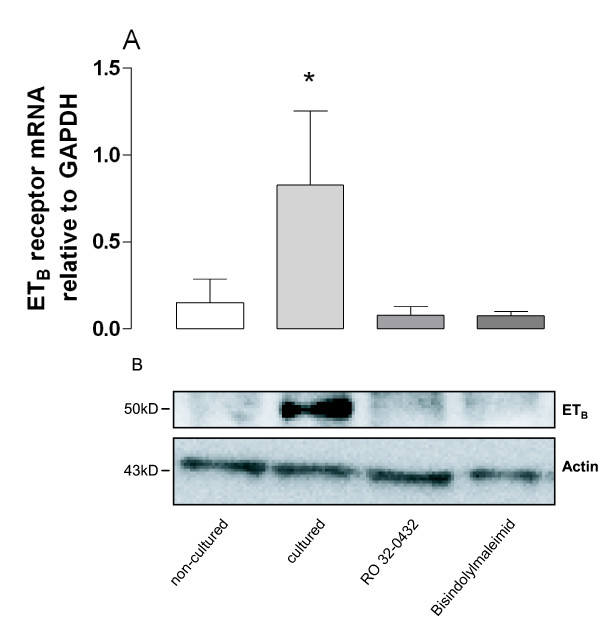
**(A) The levels of endothelin ET_B _receptor mRNA expression in human internal mammary arteries, examined using real time PCR.** (B) Endothelin ET_B _receptor protein expression in human internal mammary arteries, examined using Western blot. The arteries were either not cultured or cultured in the absence or presence of the protein kinase C inhibitors Ro-32-0432 or bisindolylmaleimide I. The results are shown as mean values ± S.E.M of six experiments. Endothelin ET_B _mRNA levels in cultured and non-cultured arteries were compared using Student's t-test, where P < 0.05 (*) was considered significant.

The efficacy of the sarafotoxin 6c-contraction was significantly increased after culture (p < 0.01, n = 27, Fig. 3) suggesting up-regulated endothelin ET_B _receptors. Similarly, Western blot and real time PCR experiments demonstrated elevated levels of endothelin ET_B _receptor protein and mRNA expression, respectively, after culture (P < 0.05, n = 6, Figure 4).

For the real time PCR experiments, similar patterns of endothelin ET_A _and ET_B _receptor mRNA expression could be shown when using β-actin as the reference gene as when using GAPDH (data not shown), indicating that these genes were trustworthy as references.

### Inhibition of PKC

The increased sarafotoxin 6c contraction and endothelin ET_B _receptor protein and mRNA expression levels during organ culture were inhibited when the arteries were cultured in the presence of the PKC inhibitors, Ro-32-0432 (10 μM) and bisindolylmaleimide I (10 μM). For results, numbers and statistics, see Fig. 3 and 4.

### Inhibition of MAPK

**Figure 5:**
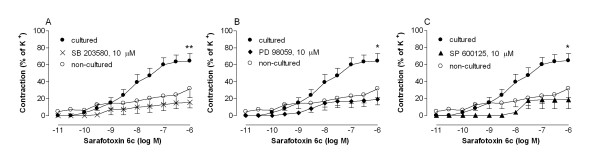
**Contractile responses elicited by cumulative application of the endothelin ET_B _receptor agonist sarafotoxin 6c in segments of human internal mammary arteries.** The arterial segments were either not cultured or cultured in the absence or the presence of mitogen-activated kinase (MAPK) pathway inhibitors; (A) the P38 MAPK inhibitor SB203580, (B) the ERK1/2 inhibitor PD98059 or (C) the JNK inhibitor SP600125. The results are shown as mean values ± S.E.M of six experiments. Statistical analysis was performed using ANOVA with Dunnett's post-test for multiple comparisons. P < 0.05 (*) and P < 0.01 (**) was considered significant. Comparisons were made between the results from arteries exposed to culture with and without SB203580, PD98059 or SP600125.

**Figure 6:**
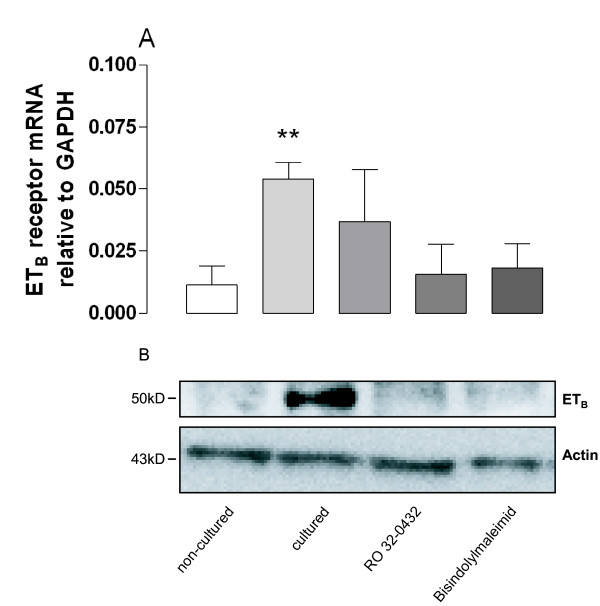
**(A) The levels of endothelin ET_B _receptor mRNA expression in human internal mammary arteries, examined using real time PCR.** (B) Endothelin ET_B _receptor protein expression in human internal mammary arteries, examined using Western blot. The arteries were either not cultured or cultured in the absence or presence of mitogen-activated kinase (MAPK) pathway inhibitors; the P38 MAPK inhibitor SB203580, the ERK1/2 inhibitor PD98059 or the JNK inhibitor SP600125. The results are shown as mean values ± S.E.M of six experiments. Endothelin ET_B _mRNA levels in cultured and non-cultured arteries were compared using Student's t-test, where P < 0.05 (*) and P < 0.01 (**) was considered significant.

The p38 MAPK pathway inhibitor, SB203580 (10 μM), the ERK1/2 pathway inhibitor, PD98059 (10 μM) and the JNK pathway inhibitor, SP600125 (10 μM), inhibited the up-regulation of sarafotoxin 6c contraction, endothelin ET_B _receptor protein and mRNA expression. For results, numbers and statistics, see Fig. 5 and 6.

## Discussion

### Main findings

Up-regulation of vascular endothelin ET_B _receptors is implicated in the pathogenesis of cardiovascular disease. This study demonstrates that the PKC and MAPK intracellular signal transduction pathways may play a role in the regulation of endothelin ET_B _receptors in the human internal mammary artery.

### Organ culture and endothelin receptor regulation

The organ culture method, used in the present study, stimulate up-regulation of endothelin ET_B _receptors in the human left internal mammary artery, as shown by *in vitro *pharmacology, Western blot and real time PCR experiments. This is in accordance with our previous findings that endothelin ET_B _receptors are up-regulated during organ culture in human coronary arteries [9]. This increase in ET_B _receptor density can be compared to that observed in arteries from patients with ischemic heart disease or hypertension [7,9,10]. Plasma levels of endothelin are elevated in ischemic heart disease and in heart failure [26,27]. Enhanced activity in the endothelin system has been associated with the progression of cardiovascular disease.

Endothelin is a strong vasoconstrictor and up-regulation of endothelin receptors on vascular smooth muscle cells causes inappropriate contraction that exacerbates atherosclerotic stenoses. Endothelin constricts human coronary arteries, especially those with atherosclerosis, and accounts for nearly all the resting tone at coronary artery stenosis [28]. Endothelin is also known to act as a mitogen on vascular smooth muscle cells, stimulate extracellular matrix synthesis and attract monocytes in the process of atherosclerosis.

We believe that it is important to gain insight into the regulation of the vascular endothelin receptor expression. Identifying the intracellular signal transduction pathways involved in the up-regulation of endothelin receptors may provide new pharmaceutical targets. Organ culture is an experimental model in which the endothelin receptor regulation can be studied in detail, *ex vivo*, to delineate the molecular mechanisms involved. Culture in the presence of different humoral factors or intracellular messenger inhibitors may reveal important pathways involved in the regulation of endothelin receptors. The method of organ culture combines the advantage of cell culturing techniques with the advantage of functional evaluation of intact blood vessels.

Increased endothelin ET_B _contraction after organ culture in the present study is presumably due to increased levels of contractile endothelin ET_B _receptors on the vascular smooth muscle cells. Previous results from our group show that the contractile response to sarafotoxin 6c in the human internal mammary artery is not significantly affected by removal of the endothelium [10]. This is in accordance with the current data since the arteries are obtained from patient with advanced coronary artery disease and probably generalized atherosclerosis and endothelium dysfunction.

### Mechanisms governing the regulation of endothelin ET_B _receptors

We do not know the exact mechanism by which the endothelin ET_B _receptors are up-regulation in culture. Previous experiments suggest that the up-regulation requires physiological oxygen and glucose levels and that the choice of buffer solution (Krebs or DMEM) does not seem to play a role [29]. The dissection procedure is similar for the non-cultured and cultured arteries and does presumably not play a role for the organ culture effects. The only difference between the non-cultured and cultured vessel segments is incubation *per se*. During incubation, perfusion pressure is lost and we speculate that this loss in sheer stress may be one factor that triggers the receptor regulation. Indeed, preliminary perfusion experiments *in vitro *revile that the regulation of endothelin ET_B _receptors is dependent on the perfusion pressure applied (not published data). Up-regulation of ET_B _receptors is known to rely on increased transcription and subsequent translation of ET_B _receptor mRNA [30]. This is in accordance with the present results that demonstrate increased levels of ET_B _receptor mRNA and protein. In the human genome, the 5'-flanking region of the genes encoding the endothelin receptors contain several regulatory elements, like GATA-motifs and E-boxes [31,32]. This indicates that the genes might be activated by for example stress related and inflammatory components.

### PKC signaling pathways

This study was aimed to elucidate the role of the PKC and MAPK signaling pathways in the endothelin ET_B _receptor regulation. The PKC antagonists, Ro-32-0432 and bisindolylmaleimide I, each inhibited the increase in sarafotoxin 6c contraction and the elevated levels of endothelin ET_B _receptor protein and mRNA expression, during organ culture. PKC signaling pathways has previously been suggested to play a role in the development of cardiovascular disease. PKC increase oxygen production in the growing atherosclerotic lesion, leading to apoptosis and plaque instability [33]. The levels of PKC in the myocardium are elevated in various models of cardiac hypertrophy [34-36]. Furthermore, PKC isozymes contribute to different stages of cardiac fibrosis [37]. Indeed, treatment with a PKCδ inhibitor has been shown to ameliorate the reperfusion injury during primary percutaneous coronary intervention for myocardial infarction [37].

### MAPK signaling pathways

The MAPK pathways are thought to act downstream from PKC in the smooth muscle cell regulatory cascade [19]. MAPKs are a family of serine/threonine kinases which are associated with vascular smooth muscle cell contraction, migration, adhesion, collagen deposition, cell growth, differentiation and survival [38]. The three major subgroups of MAPK are p38, ERK1/2 and JNK [39]. In the present study we found that the p38 MAPK pathway inhibitor, SB203580 (10 μM), the ERK1/2 pathway inhibitor, PD98059 (10 μM) and the JNK pathway inhibitor, SP600125 (10 μM), blocked the up-regulation of the endothelin ET_B _receptors in human internal mammary arteries during organ culture. This is in accordance with previous studies that have a role for MAPK pathways in cardiovascular disease. JNK is a stress-activated protein kinase while ERKs mediate cellular responses initiated by growth factors. The P38 MAPK pathway is activated by inflammatory cytokines such as TNF-α, IL-1 and IL-8, which are known to be increased in atherosclerosis and ischemic heart disease. Since the vessels were obtained from severely diseased patients the current data may suggest that there is activation of all three major MAPKs in advanced cardiovascular disease.

### Clinical relevance

Endothelin induces strong vasoconstriction in human blood vessels and endothelin receptors are up-regulated in cardiovascular disease such as hypertension, arteriosclerosis and myocardial infarction. Whether endothelin receptor antagonists will become part of the therapeutic armamentarium in hypertension and associated cardiovascular disease remains unclear. However, none of these agents is currently being developed for this indication. New endothelin antagonists devoid of side effects or alternative inhibitors of the endothelin converting enzymes may in the future become available to block the endothelin system [40]. Along with previous reports [16-18,41], the present study shows that the endothelin dependent vascular contraction and remodeling seem to be dependent on both PKC and MAPK. Inhibition of these intracellular signal tran sduction pathways may become a future approach for targeting the endothelin system in the prevention of the development of cardiovascular disease.

## Conclusion

The present findings demonstrate up-regulated endothelin ET_B _receptors in human left internal mammary arteries after organ culture, which is similar to the changes that occur in cardiovascular disease. The intracellular signal transduction pathways PKC and p38 MAPK seems to be involved in the endothelin ET_B _receptor regulation. Inhibiting these intracellular signal transduction pathways may provide future therapeutic targets for hindering the development of vascular endothelin receptor changes in cardiovascular disease.

## Competing interests

The authors declare that they have no competing interests.

## Authors' contributions

DN helped in planning the experiments, performed the in vitro pharmacology and real time PCR experiments, analyzed the data and wrote the manuscript. LG performed the immunofluorescence experiments and reviewed the manuscript. AW helped in performing the in vitro pharmacology and real time PCR experiments and the analysis of the results. BG helped in performing the real time PCR experiments and analysis of the results. LE helped in planning the project and writing the manuscript. PP performed the surgical procedure and reviewed the manuscript. RI performed the surgical procedure and reviewed the manuscript. MM conceived the study, guided the experimental procedure and helped in writing ht manuscript.

## Pre-publication history

The pre-publication history for this paper can be accessed here:


